# Increased angiotensin II coupled with decreased *Adra1a* expression enhances cardiac hypertrophy in pregnancy-associated hypertensive mice

**DOI:** 10.1016/j.jbc.2023.102964

**Published:** 2023-02-02

**Authors:** Jun-Dal Kim, Chulwon Kwon, Kanako Nakamura, Naoto Muromachi, Haruka Mori, Shin-ichi Muroi, Yasunari Yamada, Hodaka Saito, Yoshimi Nakagawa, Akiyoshi Fukamizu

**Affiliations:** 1 Life Science Center for Survival Dynamics, Tsukuba Advanced Research Alliance, University of Tsukuba, Tsukuba, Ibaraki, Japan; 2 Division of Complex Bioscience Research, Department of Research and Development, Institute of National Medicine, University of Toyama, Toyama, Japan; 3 AMED-CREST, Japan Agency for Medical Research and Development, Chiyoda-ku, Tokyo, Japan; 4 Graduate School of Sciences and Technology, University of Tsukuba, Tsukuba, Ibaraki, Japan; 5 Doctoral Program in Life and Agricultural Sciences, University of Tsukuba, Tsukuba, Ibaraki, Japan; 6 International Institute for Integrative Sleep Medicine (WPI-IIIS), University of Tsukuba, Tsukuba, Ibaraki, Japan

**Keywords:** adrenergic receptor, angiotensin II, cardiac hypertrophy, pregnancy, hypertension, ACE, angiotensin-converting enzyme, ADRA1, alpha-1 adrenergic receptors, Adra1a, alpha-1A adrenergic receptor, Ang I, angiotensin I, Ang II, angiotensin II, AT1, Ang II type 1, CREB, cAMP response element-binding protein, DEG, differentially expressed gene, ERK, extracellular signal-regulated kinase, FC, fold change, FDR, false discovery rate, FS, fractional shortening, GO, gene ontology, hAG, transgenic human angiotensinogen, HDP, hypertensive disorders during pregnancy, HR, heart rate, hRN, transgenic human renin, KEGG, Kyoto Encyclopedia of Genes and Genomes, PAH, pregnancy-associated hypertensive, PCA, principal component analysis, RAS, renin-angiotensin system, RNA-seq, RNA-sequencing

## Abstract

Cardiac hypertrophy is a crucial risk factor for hypertensive disorders during pregnancy, but its progression during pregnancy remains unclear. We previously showed cardiac hypertrophy in a pregnancy-associated hypertensive (PAH) mouse model, in which an increase in angiotensin II (Ang II) levels was induced by human renin and human angiotensinogen, depending on pregnancy conditions. Here, to elucidate the factors involved in the progression of cardiac hypertrophy, we performed a comprehensive analysis of changes in gene expression in the hearts of PAH mice and compared them with those in control mice. We found that alpha-1A adrenergic receptor (*Adra1a*) mRNA levels in the heart were significantly reduced under PAH conditions, whereas the renin-angiotensin system was upregulated. Furthermore, we found that *Adra1a*-deficient PAH mice exhibited more severe cardiac hypertrophy than PAH mice. Our study suggests that *Adra1a* levels are regulated by renin-angiotensin system and that changes in *Adra1a* expression are involved in progressive cardiac hypertrophy in PAH mice.

Pregnancy can be a risk factor for the development of cardiovascular diseases because the progression of gestation results in hemodynamic changes with increases in heart rates (HRs) and circulating blood volume. These alterations during normal pregnancy induce physiological cardiac hypertrophy (1, 2). Moreover, hypertensive disorders during pregnancy (HDP), including chronic or gestational hypertension, preeclampsia, and eclampsia, are life-threatening conditions with an incidence of 5 to 10% of all pregnancies (3, 4). Although HDP causes hypertension, proteinuria, and left ventricular hypertrophy in females, only few studies have focused on pathological cardiac hypertrophy.

The renin-angiotensin system (RAS) is involved in the regulation of blood pressure and is strongly implicated in various cardiovascular diseases (5, 6). In the RAS pathway, angiotensin I (Ang I) is produced by the reaction of renin with angiotensinogen, and angiotensin-converting enzyme (ACE) converts Ang I to angiotensin II (Ang II), a key bioactive peptide for blood pressure regulation (7). Moreover, RAS pathway is linked to pathological processes of HDP (8, 9, 10, 11). Cardiac Ang II does not affect the phenotype of the heart under normal conditions but worsens ventricular hypertrophy and fibrosis with increased blood pressure (12). However, the relationship between RAS and cardiac hypertrophy in HDP remains unclear.

Previously, we generated a HDP animal model, comprising of pregnancy-associated hypertensive (PAH) mice, by mating females expressing the transgenic human angiotensinogen (*hAG*) gene with males expressing the transgenic human renin (*hRN*) gene (13). PAH mice have phenotypes including maternal hypertension, proteinuria, and intrauterine growth retardation of embryos due to the reaction of fetal hRN and maternal hAG during late pregnancy (14, 15). These mice also exhibit pathological cardiac hypertrophy with fibrosis and apoptosis progression before delivery (16). Interestingly, treatment with Ang II receptor inhibitors improves cardiac hypertrophy in PAH mice (17). Earlier, we also showed that inhibitors of either ACE or Ang II type 1 (AT1) receptors efficiently alleviated pathological progression in the hearts of PAH mice (16, 18). Thus, the RAS pathway may play an important role in pathological cardiac hypertrophy in PAH mice and patients with HDP. However, the detailed mechanisms underlying hypertrophic progression in PAH mice remain unknown.

To better understand the molecular mechanism underlying cardiac hypertrophic progression in HDP pathology, we studied changes in cardiac gene expression in PAH mice using bulk RNA-sequencing (RNA-seq) and observed decreased alpha-1A adrenergic receptor (*Adra1a*) expression in the hearts of PAH mice. Furthermore, we showed that reduced *Adra1a* expression is critical for Ang II-induced cardiac hypertrophy in PAH mice. Thus, our data provide a link between cardiac gene expression during hypertrophic progression and gestational hypertension. This study also showed that RAS pathway inhibition has therapeutic potential in cardiac hypertrophy through its ability to reduce the alteration of *Adra1a* expression in patients with HDP.

## Results

### Transcriptomic changes in PAH mouse hearts during late gestation

**Figure 1:**
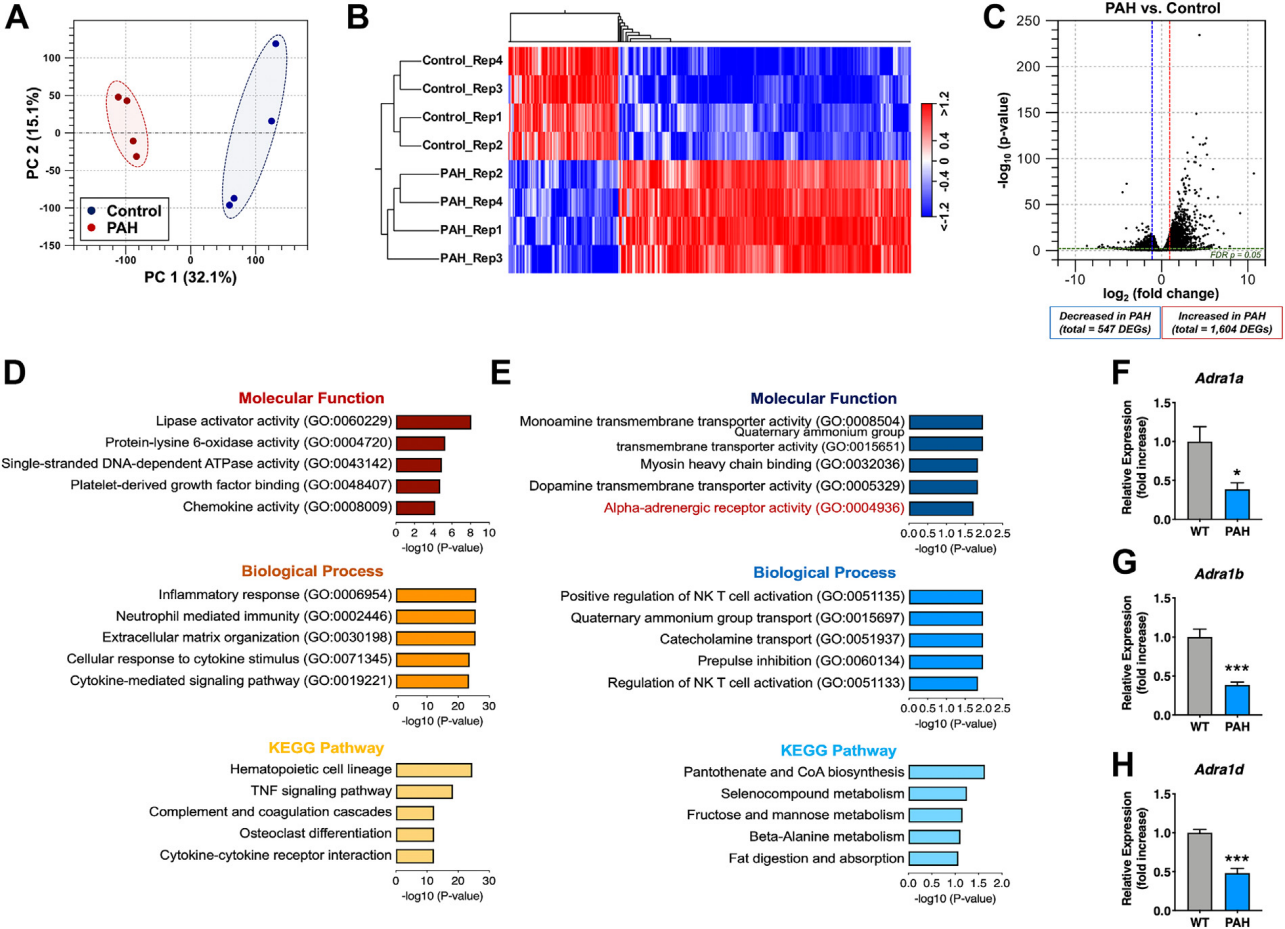
**Transcriptome analysis of PAH mouse hearts.***A*, principal component analysis of the RNA-seq data. Principal component 1 (PC1, x-axis) and PC2 (y-axis) represent 32.1% and 15.1% of the total variation, respectively. *B*, hierarchical clustering analysis showing differential gene expression between the PAH and control (WT) groups. *Red* indicates high gene expression, and *blue* shows reduced gene expression map. *C*, volcano plot showing differentially expressed genes (DEGs) between the two groups (fold change −2 to 2 filtering; FDR *p* < 0.05). *D* and *E*, gene ontology enrichment including molecular function (*top*) and biological process (*middle*), and Kyoto Encyclopedia of Genes and Genomes pathway (*bottom*) analyses between PAH and control groups for 1604 upregulated DEGs (*D*) and 547 downregulated DEGs (*E*). *F–H*, quantitative real-time PCR validation of alpha-adrenergic receptor genes; *Adra1a* (*F*), *Adra1b* (*G*), and *Adra1d* (*H*). Results are presented as the mean ± SEM; ∗*p* < 0.05, and ∗∗∗*p* < 0.001 *versus* wildtype group (unpaired *t* test). *Adra1a*, alpha-1A adrenergic receptor; GO, Gene ontology; KEGG, Kyoto Encyclopedia of Genes and Genomes; PAH, pregnancy-associated hypertensive; RNA-seq, RNA-sequencing.

To determine the alteration of gene expression in the hypertrophied hearts of PAH mice, we used RNA-seq to analyze the transcriptome in the heart tissues of wildtype (WT) and PAH mice on day 19 of pregnancy (P19). Principal component analysis (PCA) and hierarchical clustering analysis were performed to assess the reproducibility of biological replicates between WT and PAH samples. Both analyses showed significantly separated clusters between the two groups (Fig. 1, *A* and *B*).

Next, we calculated the difference in reads per kilobase per million reads, and differentially expressed genes (DEGs) were identified using a false discovery rate (FDR) <0.05, and a fold change (FC) ≥2 or ≤−2. In total, 2151 DEGs were identified, including 1604 upregulated and 547 downregulated genes in PAH hearts (Fig. 1*C* and Table S1). To further clarify the pathways significantly enriched in DEGs, we performed gene ontology (GO) and Kyoto Encyclopedia of Genes and Genomes (KEGG) pathway analyses using online-based Enrichr Suite software (19). In the classification of 1604 upregulated DEGs based on the biological process, the top five terms identified were “Cytokine-mediated signaling pathway”, “Cellular response to cytokine stimulus”, “Extracellular matrix organization”, “Neutrophil-mediated immunity”, and “Inflammatory response”, Figure 1*D*. Moreover, KEGG pathway analysis showed the terms “Cytokine-cytokine receptor interaction”, “Osteoclast differentiation”, “Complement and coagulation cascades”, “TNF signaling pathway,” and “Hematopoietic cell lineage”. These results suggest that the increased expression of fibrosis- and inflammation-associated genes positively regulates pathological cardiac hypertrophy in PAH mice during the late gestation period.

Subsequently, we performed GO and KEGG pathway analyses of the downregulated DEGs. The top five molecular function terms were collected (Fig. 1*E*). Interestingly, molecular function mapping of the DEGs revealed a highly significant association with alpha-adrenergic receptor activity. Next, to validate the results obtained using GO analysis, we determined the changes in expression of alpha- and beta-adrenergic receptor genes, including *Adra1a*, *Adra1b*, *Adra1d*, *Adrb1*, *Adrb2*, and *Adrb3* using quantitative reverse transcription–PCR. As expected, the expression of three alpha 1-adrenergic receptor genes, *Adra1a*, *Adra1b*, and *Adra1d*, was significantly decreased in PAH mice as compared to that in pregnant WT mice (Fig. 1, *F*–*H*), but not beta-adrenergic receptor genes (Fig. S1, *A*–*C*), indicating that the alpha 1-adrenergic receptor subtype genes are selectively downregulated.

### Suppression of cardiac Adra1a mRNA expression by increasing Ang II levels in pregnant mice

**Figure 2:**
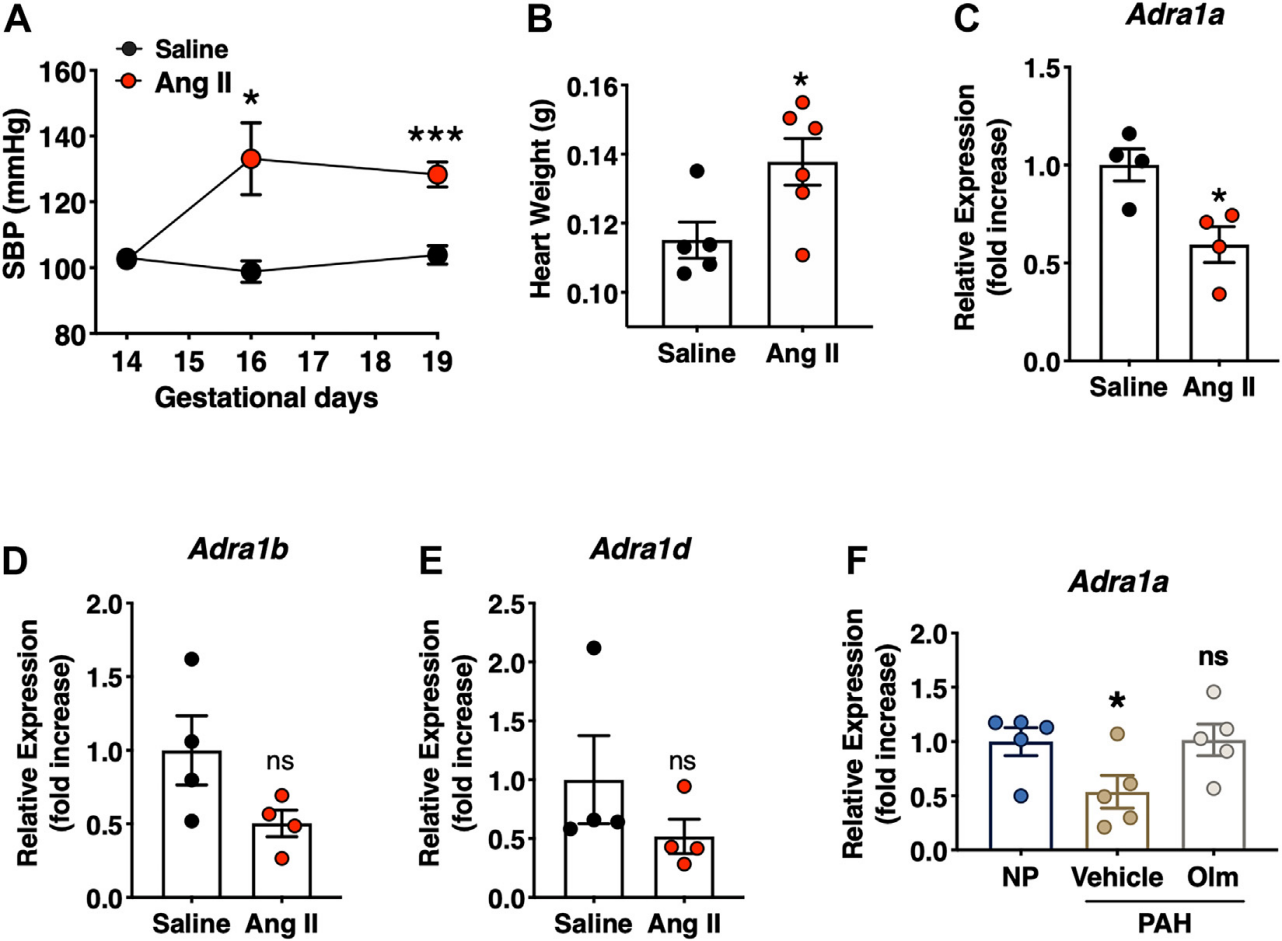
**Downregulated *Adra1a* expression in Ang II-infused pregnant mice.***A*, temporal change in systolic blood pressure of pregnant mice (n = 5 per group) with saline or Ang II (2 mg/kg/day) for 7 days. Results are presented as the mean ± SEM of the number of animals; ∗*p* < 0.05, and ∗∗∗*p* < 0.001 *versus* the saline control group (two-way ANOVA followed by Bonferroni’s post hoc test. *B*, weight of hearts of saline- or Ang II-infused mice on day 19 of pregnancy (n = 5–6 per group). *C–E*, qPCR validation for genes of alpha-adrenergic receptor genes; *Adra1a* (*C*), *Adra1b* (*D*), and *Adra1d* (*E*). *F*, levels of *Adra1a* expression in nonpregnant (NP, hAG expressing females), PAH (P19 PAH), and Olm-treated PAH (Olm PAH) mice at P19 (n = 5 per group). Results are present as the mean ± SEM; ∗*p* < 0.05, and ns (not significant) *versus* saline control group (unpaired *t* test). *Adra1a*, alpha-1A adrenergic receptor; Ang II, angiotensin II; *hAG*, transgenic human angiotensinogen; PAH, pregnancy-associated hypertensive; SBP, systolic blood pressure.

Both Ang II and ADRA1A receptor pathways play an important role in cardiovascular homeostasis (20, 21). ADRA1A is a member of the G protein-coupled receptor family that activates the phosphatidylinositol-calcium second messenger system (22). However, *Adra1a* mRNA is suppressed by Ang II in cardiomyocytes (23). Moreover, plasma Ang II levels excessively increase in PAH mice during late pregnancy (13). Thus, we focused on the relationship between Ang II and *Adra1a* expression in cardiac hypertrophy and investigated whether elevated Ang II levels regulate *Adra1a* mRNA levels during pregnancy. Ang II was infused into WT mice from day 14 of pregnancy using osmotic pumps, and systolic blood pressure (SBP) and heart weight were found to increase by day 19 of pregnancy (Fig. 2, *A* and *B*). Furthermore, although the expression of other alpha1-subtypes did not change, cardiac *Adra1a* expression was significantly decreased in Ang II-infused pregnant mice (Fig. 2, *C*–*E*).

To explore the effect of the Ang II-mediated AT1 receptor pathway on the levels of cardiac *Adra1a* mRNA in pregnant mice, we also examined the expression of *Adra1a* in nonpregnant hAG females, PAH, and RAS pathway-inhibited PAH mice. *Adra1a* mRNA levels were downregulated in the hearts of PAH mice compared to those in the nonpregnant mice, and they were recovered by administration of an AT1 receptor blocker, olmesartan (Fig. 2*F*). Taken together, these results suggest that the Ang II-AT1 receptor pathway modulates *Adra1a* mRNA levels in the hearts of PAH mice.

### Effect of Adra1a levels on cardiac hypertrophy in PAH conditions

**Figure 3:**
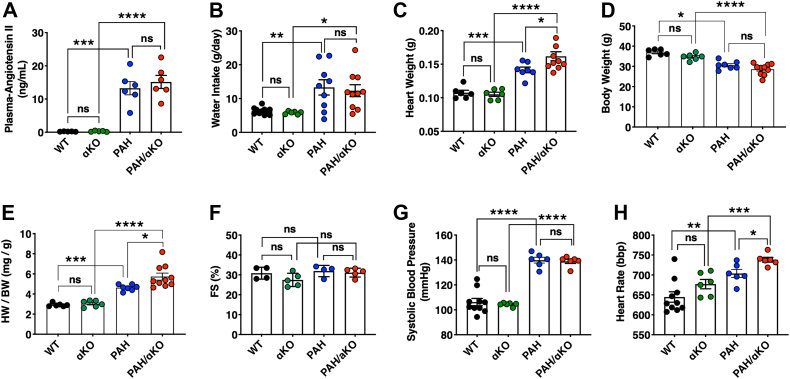
**Effect of *Adra1a* deficiency on cardiac function under PAH conditions.***A–E*, plasma levels of Ang II (*A*), water intake during 24 h (*B*), heart weight (*C*), body weight (*D*), and heart weight to body weight ratio (*E*) in WT, αKO, PAH, and PAH/αKO groups (n = 5–10 per group) at P19. *F–H*, cardiac functions of *Adra1a*-deficient PAH mice. Percentages of the left ventricular fractional shortening (*F*), systolic blood pressure (*G*), and heart rate (*H*) in four groups (n = 4–10 per group) at P19. Results are present as the mean ± SEM of the number of animals; ∗*p* < 0.05, ∗∗*p* < 0.01, ∗∗∗*p* < 0.001, ∗∗∗∗*p* < 0.0001, and ns (not significant). Statistical significance was evaluated using one-way analysis of variance using Bonferroni’s multiple comparison test. *Adra1a*, alpha-1A adrenergic receptor; Ang II, angiotensin II; BW, body weight; FS, fractional shortening; HW, heart weight; PAH, pregnancy-associated hypertensive.

To directly elucidate the effect of *Adra1a* expression levels in the hearts of PAH mice, we generated PAH/Adra1a KO (PAH/αKO) mice by mating hRN transgenic male mice with Adra1a-deficient hAG transgenic female mice (Fig. S2). Plasma Ang II levels, water intake, and heart weight of PAH mice were greater than those of WT mice, whereas these parameters were similar between PAH and PAH/αKO mice (Fig. 3, *A*–*C*). In addition, *Adra1a* deletion did not influence cardiac contraction or SBP on day 19 of gestation (Fig. 3, *D* and *E*). In contrast, the HR of the PAH/αKO mice was significantly higher than that of the PAH mice (Fig. 3*F*). These results indicate that *Adra1a* deficiency affects the HR but does not influence cardiac contractility or blood pressure under PAH conditions.

**Figure 4:**
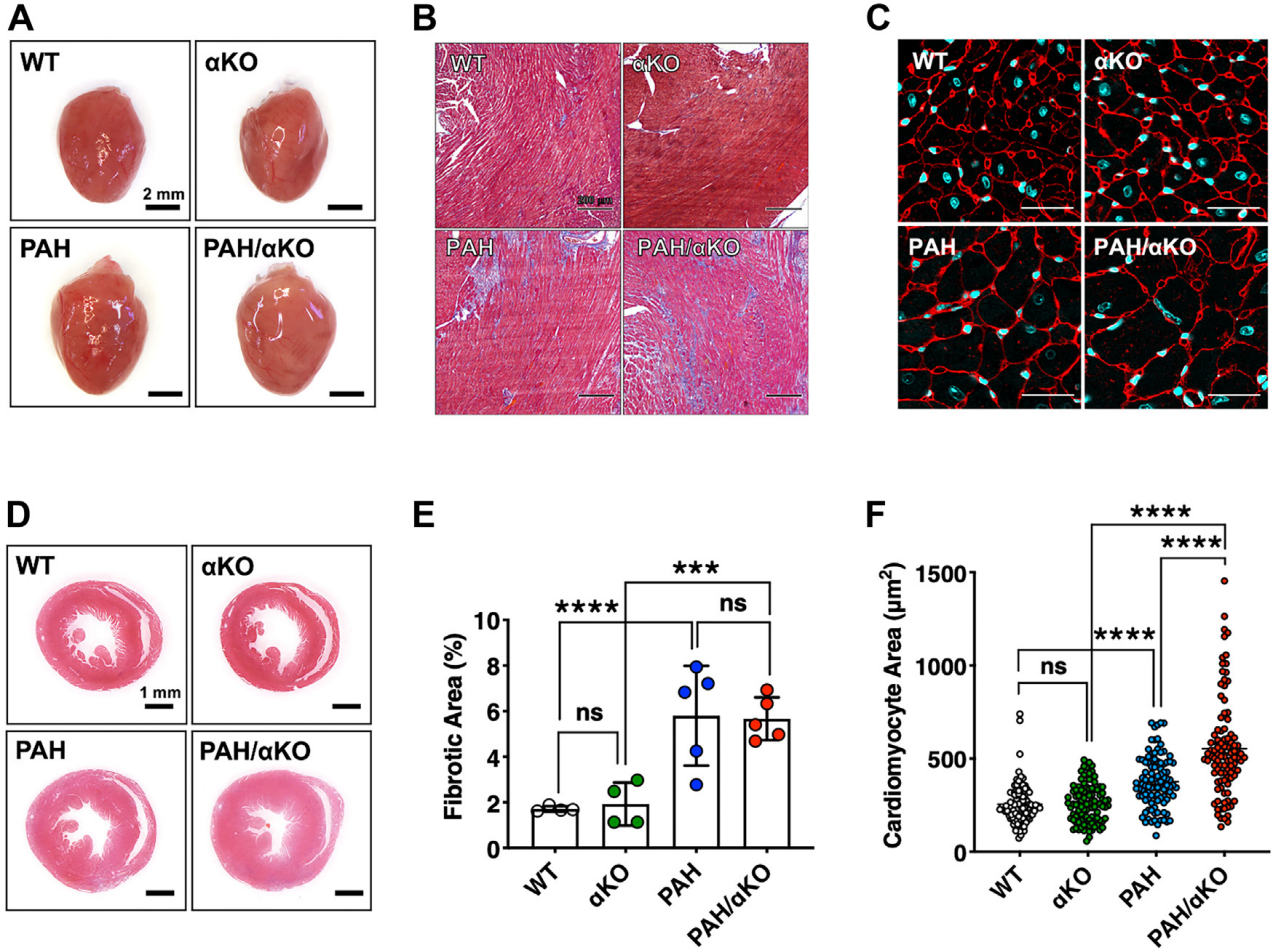
**Effect of *Adra1a* deficiency on cardiac hypertrophy under PAH conditions.***A* and *B*, heart morphology, representative images of the whole hearts (*A*) and H&E staining of sections (*B*) from each group at P19. Scale bars represent 2 mm (*A*) and 200 μm (*B*). *C* and *D*, Masson’s trichrome-stained left ventricular sections for detecting myocardial fibrosis in each group at P19 (*C*), and quantification of fibrotic areas (n = 4–5 per group) (*D*). The scale bars represent 30 μm (*C*) and 1 mm (D). *E* and *F*, representative images of cardiomyocytes in cardiac sections using fluorescently labeled wheat germ agglutinin (*E*), and quantification of cardiomyocyte areas (n = 100 per group) (*F*). Results are present as the mean ± SEM of the number of animals; ∗∗∗*p* < 0.001, ∗∗∗∗*p* < 0.0001, and ns (not significant). Statistical significance was evaluated using one-way analysis of variance using Bonferroni’s multiple comparison test. *Adra1a*, alpha-1A adrenergic receptor; PAH, pregnancy-associated hypertensive.

We investigated the effect of *Adra1a* deficiency on cardiac morphology in mice with PAH. Macroscopic and histological analyses revealed that compared with PAH mice on gestational day 19, PAH/αKO mice had cardiac hypertrophy and enlarged cardiac chambers (Fig. 4, *A* and *B*), whereas no evident difference was observed in myocardial fibrosis between the two groups (Fig. 4, *C* and *D*). To determine the cause of weight gain and increased HR in PAH/αKO mice, we investigated cardiomyocyte size using wheat germ agglutinin staining. The cross-sectional area of cardiomyocytes was significantly increased in PAH/αKO mice compared with that in PAH mice, although the fibrotic area was unchanged (Fig. 4, *E* and *F*). These data indicate that the downregulation of *Adra1a* expression aggravates cardiac hypertrophy in PAH mice.

### Identification of cardiac hypertrophy-related genes in PAH/αKO mice

**Figure 5:**
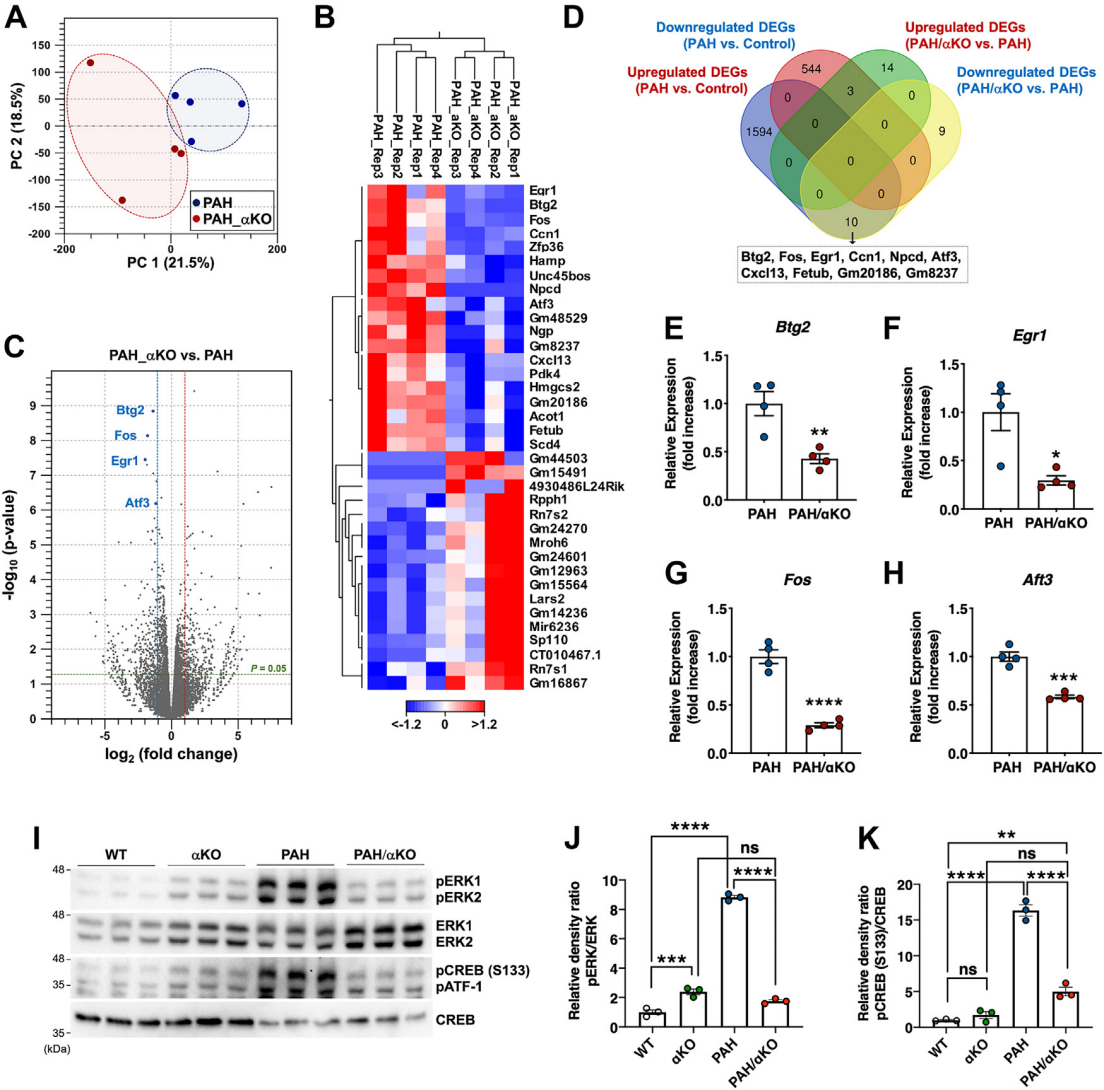
**Comprehensive analysis of transcriptome between PAH/αKO and PAH mice.***A*, PCA of RNA-seq data. PC1 (x-axis) and PC2 (y-axis) represent 21.5% and 18.5% of the total variation, respectively. *B* and *C*, hierarchical clustering (*B*) and volcano plot (*C*) analyses showing 36 DEGs between PAH/αKO and PAH groups. *D*, Venn diagram showing the overlap of DEGs among control, PAH, and PAH/αKO groups. Overlapped transcripts between the upregulated DEGs of PAH *versus* control groups and the downregulated DEGs of PAH/αKO *versus* PAH groups are highlighted in the box. *E–H*, expression levels of *Btg2* (*E*), *Egr1* (*F*), *Fos* (*G*), and *Atf3* (*H*) in the hearts of PAH and PAH/αKO mice (n = 4 per group). Results are presented as the mean ± SEM; ∗*p* < 0.05, ∗∗*p* < 0.01, ∗∗∗*p* < 0.001, and ∗∗∗∗*p* < 0.0001 *versus* control group (unpaired *t* test). *I*, representative Western blot of phospho-ERK1/2 and phospho-CREB in hearts of WT, αKO, PAH and PAH/αKO groups (n = 3 per group). Total ERK1/2 or CREB expression was used as a control. *J*, phospho-ERK1/2 signals were normalized with total ERK1/2 signals. *K*, phospho-CREB signals were normalized with total CREB signals. Results are present as the mean ± SEM of the number of animals; ∗∗*p* < 0.01, ∗∗∗*p* < 0.001, ∗∗∗∗*p* < 0.0001, and ns (not significant). Statistical significance was evaluated using one-way analysis of variance using Bonferroni’s multiple comparison test. DEGs, differentially expressed genes; PAH, pregnancy-associated hypertensive; PCA, principal component analysis; RNA-seq, RNA-sequencing.

To further identify the alteration in cardiac mRNA expression in PAH/αKO mice, we performed a comprehensive analysis of transcripts using RNA-seq datasets of PAH and PAH/αKO mice. The PCA plot showed that PAH/αKO mice were partially separated from the PAH group (Fig. 5*A*). Filtering by FC >2 (FDR *p* < 0.05) was used to identify DEGs. Based on the above terms, 36 DEGs (19 upregulated and 17 downregulated) were identified in PAH/αKO mice (Fig. 5, *B* and *C* and Table S1). Subsequently, to determine the correlation between gene expression and *Adra1a* deficiency under PAH conditions, we compared 2151 DEGs (1604 upregulated and 574 downregulated DEGs in PAH *versus* control groups) with the above 36 DEGs in PAH/αKO *versus* PAH groups. The Venn diagram for this comparison showed that *Btg2*, *Fos*, *Egr1*, *Ccn1*, *Npcd*, *Atf3*, *Cxcl13*, *Fetub*, *Gm20186*, and *Gm8237* overlapped between the upregulated DEGs of the PAH *versus* control groups and the downregulated DEGs of the PAH/αKO *versus* PAH groups (Fig. 5*D*). Notably, downregulation of *Btg2*, *Egr1*, *Fos*, or *Atf3* enhances cardiomyocyte hypertrophy (24, 25, 26, 27). Based on the results of the above comprehensive analysis, we also verified the FCs in the expression of the four cardiac hypertrophy-related genes using quantitative reverse transcription–PCR. Compared with PAH mice, the expression of these genes was significantly reduced in the hearts of PAH/αKO mice, Figure 5, *E*–*H*.

*Btg2*, *Egr1*, *Fos*, and *Atf3* are transcribed by activating the transcription factor cAMP response element-binding protein (CREB) (28). CREB is activated by extracellular signal-regulated kinase (ERK)1/2 *via* phosphorylation at serine 133 (29). ERK activation is driven by ADRA1A signaling in cardiomyocytes (30). Thus, to evaluate whether changes in *Adra1a* expression affect the activation of ERK and CREB under PAH conditions, the phosphorylation levels of ERK1/2 and CREB were determined in the hearts of PAH and PAH/αKO mice using Western blot analyses. The results demonstrated that phosphorylation levels of ERK1/2 and CREB were markedly decreased in the *Adra1a*-deficient conditions as compared with those seen in PAH mice (Fig. 5*I*). Taken together, these data indicate that ERK1/2 and CREB are potential players downstream of ADRA1A signaling in the hearts of PAH mice and that expression levels of *Btg2*, *Egr1*, *Fos*, and *Atf3* are reduced *via* Ang II-mediated downregulation of *Adra1a* in PAH conditions.

## Discussion

Pregnancy is a dynamic process linked to significant physiological changes in the cardiovascular system. For example, plasma volume and cardiac output increase by 40 to 50% during pregnancy as compared to those in nonpregnant females. These changes are necessary to ensure a continuous supply of essential nutrients and oxygen to the developing fetus (31). Pressure overload in the heart causes physiological cardiac hypertrophy in pregnant women, which is not accompanied by cardiac injury, and returns to prepregnancy levels following delivery (32, 33). In contrast, pathological conditions such as HDP are characterized by fibrosis, apoptosis, and cardiac hypertrophy, which might progress to heart failure (34, 35). However, the detailed molecular mechanisms and factors involved in hypertrophic progression of the heart in HDP are not yet elucidated.

We previously generated PAH model that exhibited pathological cardiac hypertrophy during late gestation (13, 16). Herein, we attempted to understand the molecular mechanisms underlying the pathogenesis of cardiac hypertrophy using mice as an HDP model. Transcriptome analysis of hearts in PAH conditions showed an increase in transcript numbers of fibrosis-, extracellular matrix-, and inflammation-associated genes driven by the RAS pathway and transition to pathological cardiac hypertrophy in late gestation (Fig. 1*D* and Table S1). In contrast to the upregulated genes, excessively increased Ang II levels caused a significant decrease in *Adra1a* expression in hypertensive pregnancy, which enhanced cardiac hypertrophy (Figs. 2*C* and 4, *A*, *B*, and *E*). Furthermore, the downregulation of *Adra1a* mRNA levels in the heart of PAH mice was recovered by the administration of an AT1 receptor blocker (Fig. 2*F*). Notably, Ang II-induced cardiac hypertrophy was significantly increased by *Adra1a* deficiency during pregnancy (Fig. S3). Alpha-1 adrenergic receptors (ADRA1s) are mediators of pathological hypertrophy in most models. However, activation of ADRA1A by highly selective agonists protects against cardiotoxicity and heart failure in animal models such as doxorubicin- and bleomycin-stimulated mice (36, 37). Moreover, ADRA1A overexpression in mice enhanced cardiac contractility but not hypertrophy (38). Therefore, our results suggest that the RAS pathway-derived ADRA1A regulatory pathway is involved in pathological cardiac hypertrophy during pregnancy.

RNA-seq analysis revealed that only 36 genes (19 upregulated and 17 downregulated) were differentially expressed between the heart tissues of PAH and PAH/αKO mice (Fig. 5, *B* and *C* and Table S1). Interestingly, we identified four genes, *Btg2*, *Egr1*, *Fos*, and *Atf3*, associated with cardiac hypertrophy, in overlapping DEGs between the upregulated DEGs in PAH *versus* control groups and the downregulated DEGs in PAH/αKO *versus* PAH groups (Fig. 5*D*). B-cell translocation gene 2 (*BTG2*) negatively regulates cardiac hypertrophy by decreasing the accumulation of cytosolic RNA (24), and increasing protein synthesis and proteolysis rate are primarily seen as a mechanism during cardiomyocyte hypertrophy (39). Moreover, in neonatal cardiomyocytes, Ang II or norepinephrine-activated ADRA1s induce the expression of *Egr1*, which encodes the transcription factor EGR1 (40). EGR1 negatively regulates the expression of cardiac sodium calcium exchanger-1 (25). Sodium calcium exchanger-1 downregulation aggravates various cardiac diseases, such as heart failure and ischemia, while its upregulation causes arrhythmias (41, 42, 43). In fact, *Egr1*-deficient mice show exacerbated catecholamine-induced cardiac damage compared with similarly treated WT mice (25). In addition, *Egr1* and the proto-oncogene *Fos* are significantly downregulated in myocardia with hypertrophic obstructive cardiomyopathy compared to those in the control group (26). Finally, mice deficient in *Atf3* which encodes transcription factor 3 (ATF3), a partner protein of FOS (44, 45), exhibited fibrosis and cardiac hypertrophy in response to pressure overload, suggesting an important transcriptional role for *Atf3* in the cardiovascular system (27). Taken together, the above studies suggest a relationship between a decrease in *Btg2*, *Egr1*, *Fos*, and *Atf3* expression levels and enhanced cardiac hypertrophy in PAH/αKO hearts compared to PAH conditions.

We found that Ang II repressed the expression of *Adra1a* mRNA in PAH mice. Moreover, downregulation of *Adra1a* expression or its reduced activity may be responsible for cardiac hypertrophy and heart failure in mice that develop hypertension due to increased Ang II during pregnancy. Although further experiments are necessary to determine the molecular mechanism by which the Ang II-mediated RAS pathway regulates *Adra1a* mRNA levels, our data showed that Ang II suppressed *Adra1a* mRNA levels under PAH conditions resulting in a decrease in ERK1/2 activity (Figs. 1*F*, 2C, and 5I). ERK1/2 is an upstream activator of CREB, which is a transcription factor for *Btg2*, *Egr1*, *Fos*, and *Atf3*.

Here, we show that *Adra1a* deficiency in PAH/αKO mice causes higher HRs than in PAH mice, Figure 3*H*. Moreover, PAH/αKO mice exhibited the cross-sectional area of cardiomyocytes that was significantly increased in PAH/αKO mice compared with that in PAH mice (Fig. 4*F*). However, it is currently unclear regarding the relationship between increased cross-sectional area of cardiomyocytes and elevated HR. It has been thought that there is no direct link between increased cross-sectional area of cardiomyocytes and elevated HR, because these two phenotypes can be influenced by multiple mechanisms, such as activated RAS system or adrenergic receptors with catecholamines. On the other hand, Ang II directly increases HRs and the cross-sectional area of cardiomyocytes in animal models (46, 47), and it is also reported that the HR of *Adra1a*-deficient mice is slightly (not significantly) higher than that of WT mice (48). Furthermore, it is suggested that ADRA1s in cardiomyocytes show a variety of beneficial cardiac effects, including physiological hypertrophy, protection from apoptosis, and increased contractility (49). Therefore, it may be possible that elevated HR and increased cross-sectional area of cardiomyocytes in hearts of PAH/αKO mice are higher than that of control or PAH mice, as a synergistic effect.

Use of RAS pathway targeting drugs (such as ACE inhibitors and ARBs) to treat HDP during first trimester is worrisome because of the risk of congenital malformations (50). Moreover, the use of a nonselective Adra1 antagonist for treating hypertension or prostate disease is concerning owing to significant adverse side effects, such as increased risk of heart failure (51). The effects of selective Adra1a antagonists on cardiac disease with RAS hyperactivity have yet to be reported. Therefore, the present study provides new insights into pathological cardiac hypertrophy in pregnant women.

## Experimental procedures

### Antibodies

Anti-phospho-p44/42 MAPK (Erk1/2) (Thr202/Tyr204) antibody (#9101), anti-phospho-CREB (Ser133) (87G3, #9198), anti-CREB (D76D11, #4820), and anti-GAPDH antibody (D1611, #5174) were purchased from Cell Signaling Technology Inc Anti-ERK1+ERK2 antibody (EPR17526, ab184699) was purchased from Abcam plc.

### Animals

All animal experiments were approved by the Institutional Animal Experiment Committee of the University of Tsukuba. All experiments were performed in accordance with the Regulation of Animal Experiments of the University of Tsukuba and the Fundamental Guidelines for Proper Conduct of Animal Experiments and Related Activities in Academic Research Institutions under the jurisdiction of the Ministry of Education, Culture, Sports, Science, and Technology of Japan. Mice were maintained in the Life Science Center for Survival Dynamics, Tsukuba Advanced Research Alliance-SPF space, at 22 °C, with humidity of 40 to 60%, and 12 h light-dark cycle. All mice had free access to commercial chow (MF diet; Oriental Yeast Co, Ltd). Food and drinking water were provided ad libitum throughout the experiment.

To develop PAH mice, hAG-expressing females were crossed with hRN-expressing males (13). The day of mating was defined as day zero of pregnancy (P0). PAH mice were orally administered olmesartan (15 mg/l; kindly gifted by Daiichi Sankyo) from P13 to P19.

Ang II-infused pregnant mice and WT female and male mice (C57BL/6J) at 10 to 12 weeks of age were purchased from Japan CLEA Co. After the day of mating, pregnant mice were administered Ang II (4001, Peptide Institute Inc) (2 mg/mouse body weight [kg]/day) using an osmotic pump (1002, Alzet) for 6 days. The control mice were infused with saline using an osmotic pump.

To generate PAH/αKO mice, *Adra1a* deficiency was induced by a frameshift mutation close to the start codon of Adra1a in the hAG-overexpressing strain. Specifically, Adra1a-specific guide oligonucleotides were annealed and inserted into the *Bbs*I-digested CRISPR-Cas9 pX330 vector (42230, Addgene). The constructs were microinjected into the pronuclei of fertilized oocytes of ICR strain female mice (Charles River Laboratories Japan) and were then transferred into pseudopregnant ICR mice to obtain the initial generation of genome-edited mice. These mice were backcrossed twice with the C57BL/6J strain (Charles River Laboratories) and mated with the hAG strains. Finally, *Adra1a*-deficient hAG females with an indel mutation (1-base insertion) close to the start codon of *Adra1a* were generated (Fig. S2). PAH/αKO mice were generated by mating hRN male mice with hAG/α1A-AR-KO female mice. Oligonucleotide sequences are listed in Table S2.

### RNA preparation and RNA-sequencing

On day 19 of gestation, the hearts of pregnant mice were removed and flash-frozen in liquid nitrogen. Frozen heart tissues were homogenized using a multi-bead shocker (Yasui Kikai). Total RNA was extracted using ISOGEN II reagent (#311-07361, Nippon Gene), according to the manufacturer’s instructions.

Approximately 500 ng of total RNA were ribosomal RNA-depleted using the NEBNext rRNA Depletion Kit (E6310, New England Biolabs) and converted to an Illumina sequencing library using the NEBNext Ultra Directional RNA Library Prep Kit for Illumina (E7420, New England Biolabs). The libraries were validated using a Bioanalyzer (Agilent Technologies) and sequenced using NextSeq 500 (Illumina) with the paired-end 36-base read option. Reads were mapped to the mm10 mouse reference genome and quantified using CLC Genomics Workbench (QIAGEN). RNA-seq datasets were deposited in the NCBI Gene Expression Omnibus database (www.ncbi.nlm.nih.gov/geo; accession number: GSE140948).

### Identification of DEGs

Reads were mapped onto the GRCm38 (mm10) mouse reference genome, and the expression patterns of transcripts were estimated between sample groups. The read counts were normalized by calculating the number of reads per kilobase per million reads for each transcript in individual samples using CLC Genomics Workbench software (version 12.0, QIAGEN). DEGs were identified using the FC (−2 to +2) filtering analysis and FDR *p* < 0.05. Then, a comparison of distinct gene expressions was visualized using the PCA plot and clustering heat map analyses. Volcano plots were used to visualize the significance of changes in gene expression between the −log_10_ *p*-value and log_2_ FC.

### Functional enrichment analysis

The web-based Enrichr suite (http://amp.pharm.mssm.edu/Enrichr) (19) was used to assign GO and KEGG pathway enrichment of DEGs between sample groups.

### Quantitative real-time PCR analysis

Total RNA from the heart tissues was quantified using a NanoDrop spectrophotometer (NanoDrop 2000, Thermo Fisher Scientific). Complementary DNA was synthesized using a QuantiTect Reverse Transcription Kit (205311, QIAGEN).

Quantitative real-time PCR was performed using a Thermal Cycler Dice and SYBR Premix Ex Taq II (RR820S, TaKaRa Bio Inc). All reactions were performed in duplicate. The PCR conditions used were as follows: one cycle for 30 s at 95 °C, followed by 40 cycles of 95 °C for 5 s, 60 °C for 30 s, followed by 95 °C for 15 s and 60 °C for 30 s, and finally one cycle for 15 s at 95 °C. Data were normalized to *Gapdh* mRNA levels and the ΔΔ*C*_t_ method was used for all comparative analyses. The primer sequences are shown in Table S3.

### Measurement of cardiac function, blood pressure, and HR

Left ventricular fractional shortening (FS) was measured by echocardiography (Vevo 2100 High-Resolution Imaging System, Visual Sonics Inc). FS percentages were calculated using the left ventricle internal diameter at end-diastole (LVIDd) and end-systole (LVIDs) using the following formula:

$$FS\left(\%\right)=\left(\frac{LVIDd-LVIDs}{LVIDd}\right)*100$$

SBP and HR were measured with a programmable sphygmomanometer (BP98A, Softron) using the tail-cuff method, as described previously (52). Before pregnancy, all mice were habituated to the measurements thrice.

### Enzyme-linked immunosorbent assay for measuring Ang II

Blood from the inferior vena cava of the mice was collected using a 27-gauge needle (NN-2719S, Terumo Co) with a 1 ml syringe (SS-01T, Terumo Co). Plasma was collected by centrifugation and stored at −80 °C. Ang II levels in the plasma were measured using an Angiotensin II ELISA Kit (ADI-900-204, Enzo Life Sciences).

### Histological analysis

Heart tissue samples were harvested and fixed in 10% formalin neutral buffer solution (062-01661, FUJIFILM Wako Pure Chemicals Corp) at 4 °C for 24 h. After dehydration, the fixed samples were embedded in paraffin. The samples were sectioned into 5 μm thickness using a rotary microtome (HM340E, Microm International GmBH) and stained with hematoxylin-eosin (H&E) using a standard laboratory protocol. For the evaluation of cardiac fibrosis, sections were stained with Masson’s trichrome stain, as described previously (53). Images were acquired using an SZ61 microscope (Olympus Life Sciences, [Evident]) or BX53 microscope (Olympus) with a DP21 digital camera (Olympus).

To measure the cross-sectional areas of cardiomyocytes, 3 μm thick heart tissue sections were deparaffinized, and the plasma membrane and nuclei were stained with CF594-conjugated wheat germ agglutinin (29023, Biotium) and Hoechst 33258 (H341, Dojindo Molecular Technologies Inc), respectively. Fluorescence images were obtained using a confocal laser-scanning microscope (Fluoview FV10i, Olympus), and staining was quantified using an image analysis ImageJ software (https://imagej.nih.gov/ij/).

### Western blotting

Frozen heart tissue was ground, and the powder was resuspended in a lysis buffer (20 mM Tris-HCl (pH 7.4) 150 mM NaCl, 5 mM EDTA, pH 8.0, 1% Nonidet P-40, 5% glycerol, 1× protease inhibitor cocktail [Nacalai Tesque Inc], and 1× phosphatase inhibitor cocktail [Nacalai Tesque]) and were lysed using a bath-type sonicator (Bioruptor UCD-250, Cosmo Bio Co Ltd). The samples were then incubated on ice for 10 min. After centrifugation at 14,000 rpm at 4 °C for 10 min, the concentration of total proteins in the supernatant was determined using the Bradford assay with Bio-Rad protein assay dye reagent concentrate (Bio-Rad Laboratories). The lysates were mixed with Laemmli sample buffer containing 100 mM DTT. Heat-denatured samples were resolved by 10% SDS-PAGE and then were transferred onto an Immobilon-P membrane (MilliporeSigma). Membranes were blocked for 45 min at room temperature with 5% skimmed milk in 1× Tris-buffered saline and Tween 20 buffer. Blots were incubated with the appropriate primary antibodies at 4 °C overnight and with secondary antibodies at room temperature for 45 min. After washing with 1× Tris-buffered saline and Tween 20 buffer, chemiluminescent signals were developed using SuperSignal West Femto Maximum Sensitivity Substrate (Thermo Fisher Scientific) and then detected by exposure to X-ray film (FUJIFILM).

### Statistical analysis

All statistical analyses were performed using GraphPad Prism 8 (GraphPad Software for Macintosh). Data are expressed as mean ± standard error of the mean (SEM). Statistical significance was determined at the ∗*p* < 0.05, ∗∗*p* < 0.01, ∗∗∗*p* < 0.001, and ∗∗∗∗*p* < 0.0001 levels using an unpaired *t* test for comparisons between two groups. For multiple comparisons, we used one-way analysis of variance with Bonferroni’s multiple comparison test or two-way analysis of variance followed by Bonferroni’s post-hoc test.

## Data availability

All data are available in this paper. All RNA-seq datasets were deposited in the NCBI Gene Expression Omnibus (GEO) database (www.ncbi.nlm.nih.gov/geo; accession number: GSE140948).

## Supporting information

This article contains supporting information.

## Conflict of interest

The authors declare that they have no conflicts of interest with the contents of this article.
