# Supplementary material for: Increased angiotensin II coupled with decreased Adra1a expression enhances cardiac hypertrophy in pregnancy-associated hypertensive mice
Source: J Biol Chem. 2023 Feb 2;299(3):102964. doi: 10.1016/j.jbc.2023.102964 (PMC10011504; doi:10.1016/j.jbc.2023.102964)
Supplement: Supporting Figures S1–S3 and Tables S2–S3 [file mmc1.pdf]

Supporting information

Increased angiotensin II coupled with decreased *Adra1a* expression enhances cardiac hypertrophy in pregnancy-associated hypertensive mice.

Jun-Dal Kim*, Chulwon Kwon, Kanako Nakamura, Naoto Muromachi, Haruka Mori, Shin-ichi Muroi, Yasunari Yamada, Hodaka Saito, Yoshimi Nakagawa and Akiyoshi Fukamizu*

*For correspondence: Jun-Dal Kim, jdkim@inm.u-toyama.ac.jp; Akiyoshi Fukamizu, akif@tara.tsukuba.ac.jp.

Figure S1 to S3

Table S1 to S3

Supporting information

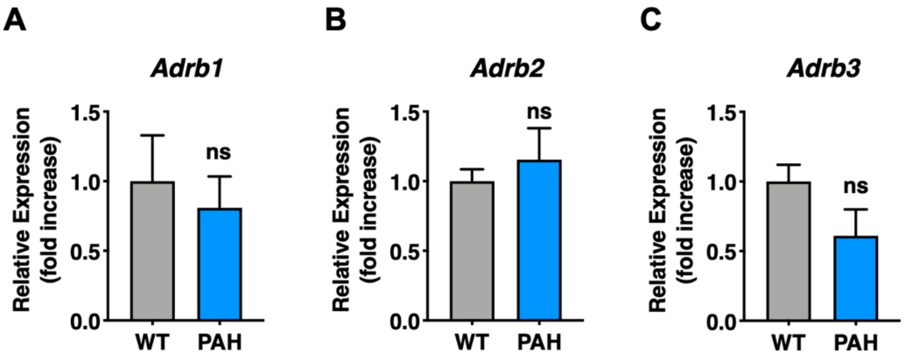

Figure S1. Quantitative real-time PCR validation for the cardiac *Adrbs* genes between WT and PAH mice. *Adrb1* (**A**), *Adrb2* (**B**), and *Adrb3* (**C**). Results are presented as the mean ± SEM of n = 4; ns (not significant) vs. control (WT) group (unpaired Student's *t*-test).

Supporting information

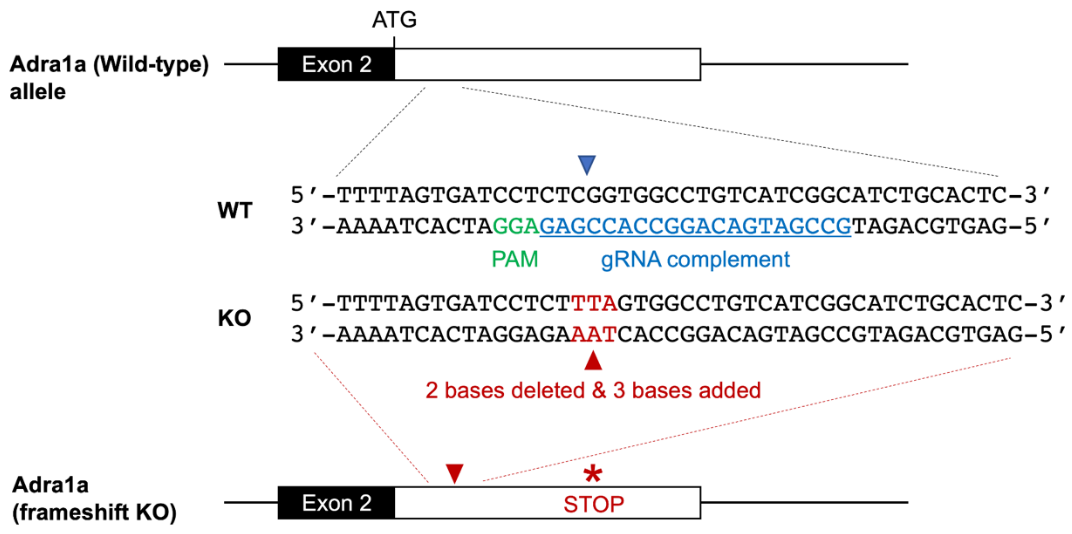

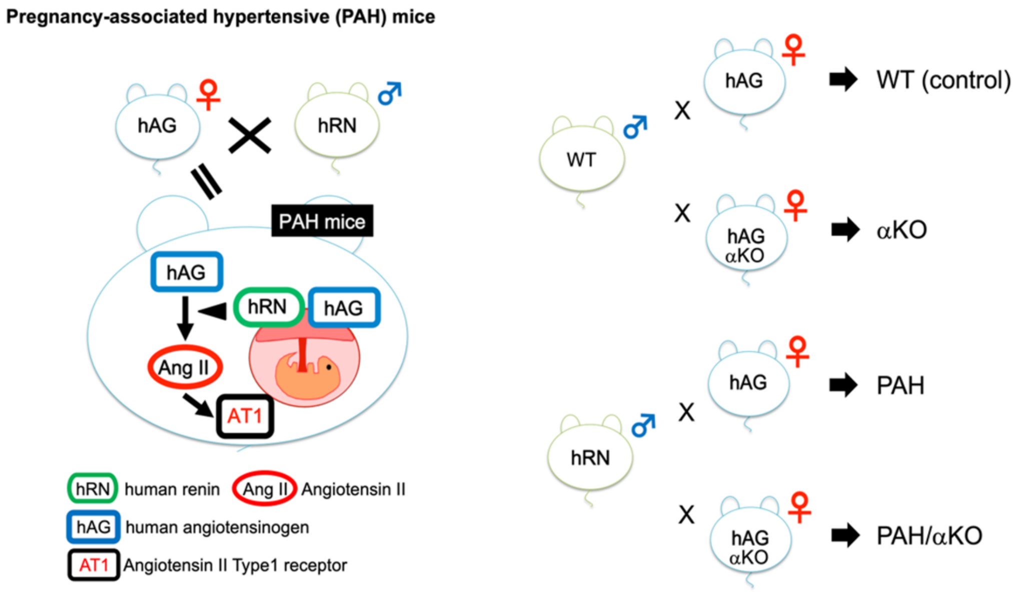

Figure S2. Schematic diagram of the α1A-AR gene targeting using genome editing with CRISPR/Cas9 system. Sequences of wild-type (PAH) and mutated (PAH/αKO) mice in target region. There were indel mutation on Adra1a target sequence, introducing a frameshift mutation that abolishes Adra1a gene function.

Supporting information

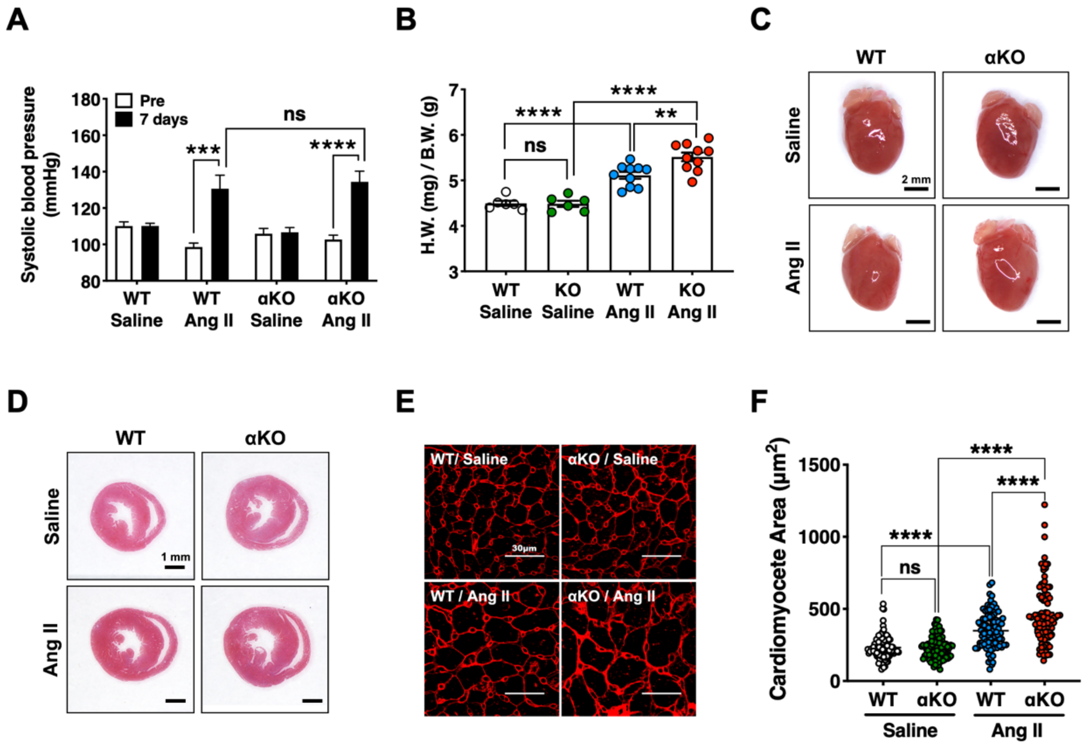

Figure S3. Effect of *Adra1a* deficiency on cardiac hypertrophy in Ang II-infused pregnant mice. A, Systolic blood pressure at P19. **B**, Heart weight (H.W.) to body weight (B.W.) ratio. **C**, **D**, Heart morphologies. Representative images of the whole hearts (C) and H&E staining of sections (D) from each group at P19. Scale bars represent 2 mm (C) and 1 mm (D). **E**, **F**, Representative images of cardiomyocytes in cardiac sections using fluorescently labeled WGA (E), and quantification of cardiomyocyte areas (n = 100 per group) (F). Scale bars represent 30 μm. Results are presented as the mean ± SEM; $^{**}P < 0.01$, $^{****}P < 0.0001$, and ns (not significant) vs. control groups. Statistical significance was evaluated using one-way ANOVA with Bonferroni's multiple comparison test.

Supporting information

Table S1. The list of differentially expressed genes (DEGs) DGEs (PAH vs. control (A) and PAH/αKO vs. PAH (B)) (Excel file).

Table S2. Oligonucleotides used in Adra1a-targeting by the CRISPR/Cas9 system.

Primer name	Sequence
Target_S	caccGCCGATGACAGGCCACCGAG
Target_AS	aaacCTCGGTGGCCTGTCATCGGC

Overhangs for ligation into the *Bbs* I site in CRISPR-Cas9 px330 vector are shown is lower case.

Table S3. The primer sequences used in qPCR.

Gene	Forward	Reverse
Adra1a	GCGGTGGACGTCTTATGCT	TCACACCAATGTATCGGTCGA
Adra1b	CCTGGTCATGTACTGCCGA	GACTCCCGCCTCCAGATTC
Adra1d	TGCAGACGGTCACCAACTATTT	GGCAACACAGCTGCACTCAG
Adrb1	CACTTTCTGCAAGGACCCGA	TCCTAGGTGTGGAACCGGAA
Adrb2	AATAGCAACGGCAGAACGGA	TCAACGCTAAGGCTAGGCAC
Adrb3	TAGGACCTGACCCTGTCATCC	TTCTAAGCCTTTCATGCCCACA
Btg2	GCACTGACCGATCATTACAAACA	CACCTTGCTGATGATGGGGT
Egr1	ACCTGACCACAGAGTCCTTTT	AGCGGCCAGTATAGGTGATG
Fos	GGCAGAAGGGGCAAAGTAGA	GATCTGTCTCCGCTTGGAGT
Atf3	GCTGCTGCCAAGTGTCGAAA	TACATGCTCAACCTGCACCG
Gapdh	TGTGTCCGTCGTGGATCTGA	TTGCTGTTGAAGTCGCAGGAG
